# Clinical Benefits of Sustained Oral Nirmatrelvir/Ritonavir Use for the Outpatient Treatment of COVID-19: Findings from the Taiwanese Health Authority Perspective Using a Decision Tree Modeling Approach

**DOI:** 10.3390/jmahp12040026

**Published:** 2024-11-12

**Authors:** Matthew Sussman, Jennifer Benner, Tendai Mugwagwa, Jackie Lee, Sheng-Tzu Hung, Ya-Min Yang, Yixi Chen

**Affiliations:** 1 Stratevi, LLC, Boston, MA 02111, USA; jennifer@stratevi.com; 2 Pfizer Ltd., Tadworth KT20 7NS, UK; tendai.mugwagwa@pfizer.com; 3 Stratevi, LLC, Santa Monica, CA 90401, USA; jackie@stratevi.com; 4 Pfizer Inc., Taipei City 110, Taiwan; alice.hung@pfizer.com (S.-T.H.); ya-min.yang@pfizer.com (Y.-M.Y.); 5 Pfizer Investment Co., Ltd., Beijing 100010, China; yixi.chen@pfizer.com

**Keywords:** coronavirus disease 2019, COVID-19, nirmatrelvir, nirmatrelvir/ritonavir, clinical burden, model, Taiwan

## Abstract

Despite the observed clinical benefits of nirmatrelvir/ritonavir (NMV/r), it is uncertain whether Taiwan will continue covering NMV/r for high-risk individuals with mild-to-moderate coronavirus disease 2019 (COVID-19). This analysis assessed the impact of sustained utilization of NMV/r on COVID-19-associated healthcare resource utilization (HCRU) and mortality from the Taiwanese health authority perspective (THAP). A decision tree model estimated the incremental number of clinical events associated with NMV/r utilization over a 30-day period. Model results compared (1) a base case using current rates of NMV/r from the THAP, and (2) a hypothetical scenario assuming the current supply of NMV/r is not extended in Taiwan. NMV/r utilization rates included 80% and 0% in the base case and hypothetical scenario, respectively. Outcomes included the number of hospitalizations involving a general ward (GW) stay, intensive care unit (ICU) stay, and mechanical ventilation (MV) use, as well as the number of bed days, symptom days, and hospitalization deaths. Based on epidemiologic data, 150,255 patients with COVID-19 were eligible for treatment from the THAP. In the hypothetical scenario, HCRU increased by 175% compared to the base case, including increases in hospitalizations involving GW, ICU, and MV use (differences: 2067; 623; 591, respectively), bed days (difference: 51,521), symptom days (difference: 51,714), and deaths (difference: 480). Findings indicate that sustained utilization of NMV/r from the THAP reduces the clinical burden of mild-to-moderate COVID-19 through the reduced incidence of COVID-19-related HCRU and deaths.

## 1. Introduction

The novel coronavirus disease 2019 (COVID-19), caused by severe acute respiratory syndrome coronavirus 2 (SARS-CoV-2), has resulted in more than 10 million cases and more than 17,000 deaths since the start of 2022 in Taiwan [1,2]. Several variants and subvariants of COVID-19 have been identified since the origination of the pandemic in December 2019. Omicron, the current predominant variant worldwide and in Taiwan [3], emerged in late 2021 and poses similar risks of progression to severe illness when compared to earlier variants [4,5,6], potentially resulting in hospitalizations, intensive care unit (ICU) stays, receipt of invasive mechanical ventilation (IMV), and/or death [7].

Taiwan has been able to mitigate many of the clinical, humanistic, and economic implications of COVID-19 that other countries have experienced, using several strategies including the closing of borders and testing and isolating. However, since April 2022, Taiwan has experienced three distinct waves of COVID-19, largely driven by an increase in the prevalence of the Omicron variant and a loosening of border restrictions, testing, and quarantine policies [8]. In the most recent wave, from December 2022 to March 2023, there were 1,910,476 cases of COVID-19 [1]. During this time, the average number of beds (either general ward or ICU) required to treat COVID-19 at a given location increased significantly, signaling a high resource use burden for hospitals. Specifically, the average number of beds required reached 577, 3423, and 1867 beds per day during the first, second, and third waves, respectively, compared to an average of 13 beds per day during non-wave periods [9]. After 20 March 2023, restrictions were lifted, and patients who were asymptomatic or had mild symptoms did not need to report their COVID-19 case or remain isolated [8].

Nirmatrelvir, a novel oral antiviral therapy, in combination with ritonavir was evaluated in the Evaluation of Protease Inhibition for COVID-19 in High-Risk Patients (EPIC-HR) clinical trial (ClinicalTrials.gov number, NCT04960202) [10]. The primary efficacy endpoint, a composite outcome defined as the reduction in COVID-19-related hospitalization or death from any cause through day 28, was met; patients with high risk of progressing to severe COVID-19 treated with nirmatrelvir/ritonavir (NMV/r) within 5 days of symptom onset had an 87.8% risk reduction of severe disease progression relative to placebo (−5.62 percentage points [95% confidence interval, −7.21 to −4.03; *p* < 0.001]) [10]. NMV/r also significantly reduced the risk of hospitalization and death. EPIC-HR assessed NMV/r in non-hospitalized adults with COVID-19 (*n* = 2113) who initiated treatment within 5 days of symptom onset. NMV/r was associated with lower rates of COVD-19 hospitalization (0.92% vs. 6.37%) and death (0% vs. 1.21%) vs. placebo when taken within 5 days of symptom onset. It showed 86% relative risk reduction (RRR) of COVID-19 related hospitalization or death by any cause if patients were treated with NMV/r within 5 days of symptom onset [11]. NMV/r has been approved by the Taiwan Food and Drug Administration (TFDA) for the treatment of individuals 12 years of age and older with mild-to-moderate coronavirus disease 2019 (COVID-19) and who are at high risk for progression to severe COVID-19 [12].

NMV/r was also evaluated in a post hoc analysis of pooled data from EPIC-HR and the Evaluation of Protease Inhibition for COVID-19 in Standard-Risk Patients (EPIC-SR) clinical trial (ClinicalTrials.gov number, NCT05011513) [13]. The EPIC-SR trial enrolled vaccinated patients with at least one risk factor for progression to severe COVID-19. In this integrated analysis of unvaccinated seropositive and vaccinated patients with at least one risk factor, NMV/r reduced the risk of COVID-19-related hospitalizations or death due to any cause by 73.8% RRR (*p* = 0.0104) compared to placebo (0.50% vs. 1.9%) within 5 days of symptom onset.

In addition to clinical trials, several real-world analyses in various geographic regions have demonstrated higher clinical effectiveness of NMV/r compared to the standard of care [14,15,16,17,18,19,20,21,22,23,24,25,26]. These real-world studies have demonstrated the robustness of NMV/r’s clinical effectiveness across both pre-Omicron- (i.e., Delta) and Omicron-variant eras of COVID-19 and vaccinated populations. Recently, Lewnard et al. conducted a matched cohort observational study of patients with and without NMV/r using electronic health records (EHRs) of non-hospitalized patients from a United States (US)-based health plan system [18]. When NMV/r was administered within 5 days of symptom onset, the primary outcome was a reduction in either COVID-19-related hospitalization or death from any cause within 30 days of a positive COVID-19 test, similarly to the clinical trials. Findings indicated that NMV/r was associated with a 79.6% reduction in hospitalizations or death (*p* < 0.0080). These findings are also supported by populations more similar to the Taiwanese perspective in size and geography [20,21,22,23].

In addition to real-world retrospective analyses, NMV/r treatment use has been analyzed via economic modeling. Specifically, a recent analysis by Bai et al. analyzed the population-level benefits of expanded clinical use of NMV/r in the treatment of COVID-19 [27]. The authors used a stochastic individual-based network model to simulate COVID-19 transmission dynamics. NMV/r treatment utilization was varied in multiple scenarios (20%, 50%, 80%, and 100%), and the authors found that increasing the treatment utilization rate was associated with higher reductions in both deaths and socioeconomic costs.

In Taiwan, government-funded access to NMV/r is currently restricted to adults ≥ 65 years of age, cancer, diabetes, chronic kidney disease, most cardiovascular disease, being pregnant or having given birth within six weeks, chronic lung disease, tuberculosis, chronic liver disease, disabilities (e.g., attention deficit hyperactivity disorder, cerebral palsy, congenital defects), mental illness, certain underlying medical conditions including obesity (body mass index [BMI] ≥ 30), and other conditions that affect the immune system [28]. Disease burden is substantial among this high-risk population. Older age is the highest risk factor for severe COVID-19 outcomes, and the number of comorbidities has an additive effect to age [29,30]. Despite the clinical benefits observed in a clinical trial environment, real-world settings, and modeling applications, there is uncertainty as to whether Taiwan will continue covering courses of NMV/r for high-risk patients. Reductions in the availability, and by extension the utilization, of NMV/r may have important clinical implications, especially given the clinical effectiveness observed in the real world and the high healthcare resource utilization (HCRU) and mortality burden associated with COVID-19-related hospitalizations. The objective of this analysis was to assess the impact of sustained utilization of NMV/r on COVID-19-associated HCRU and mortality among high-risk vaccinated and unvaccinated adult patients from the Taiwanese health authority perspective using a decision tree modeling approach.

## 2. Materials and Methods

### 2.1. Model Overview

A flexible decision tree model was designed based on prior economic evaluations of inpatient or outpatient treatments for COVID-19, including a budget impact model of NMV/r from the US perspective [31,32,33,34]. The target population for analysis consisted of non-hospitalized adults diagnosed with symptomatic COVID-19 who were at high risk of progression to severe disease, consistent with the clinical trials of NMV/r [10,13]. The model was conducted from the Taiwanese health authority perspective and was estimated based on epidemiological and clinical data from the Omicron variant era.

The model estimated the incremental number of clinical events associated with utilization of NMV/r compared to usual care. The model time horizon was set to 30 days in an effort to simulate the acute period of a COVID-19 infection and to evaluate the short-term impact of NMV/r [10,18]. Model input values were sourced from public epidemiological sources, the EPIC-HR study, and other real-world data (RWD) analyses of patients diagnosed with COVID-19 [1,11,18,35,36,37,38,39,40,41,42,43,44,45,46,47,48,49]. All model input parameters and values are provided in Table 1. Model calculations were executed in Microsoft^®^ Excel for Windows, version 2401 [50].

### 2.2. Treatment Utilization of Nirmatrelvir/Ritonavir

Results of the model compared two scenarios: (1) a base case scenario using current rates of NMV/r from the Taiwanese health authority perspective, and (2) a hypothetical scenario based on a reduced rate of NMV/r assuming the current supply of NMV/r is not extended in Taiwan. The base case of the model employed a utilization rate for NMV/r of 80%, while the hypothetical scenario assumed a utilization rate for NMV/r of 0% to align with a lack of availability of NMV/r if current stockpiling strategies are not maintained. In one-way sensitivity analyses (OWSA), the utilization rate for NMV/r in the hypothetical scenario was adjusted to account for potential variations in this model input, as explained in more detail below.

### 2.3. Model Structure

**Figure 1:**
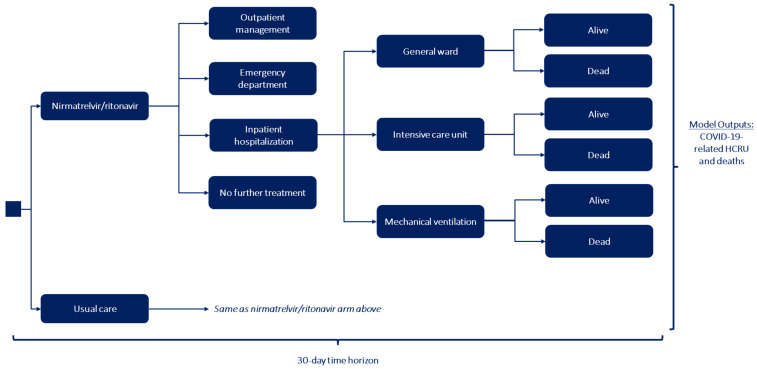
COVID-19 Model Schematic.

The model schematic is presented in Figure 1. At model start, patients were assumed to have been diagnosed in the outpatient setting. The model population was assumed to be a blend of both vaccinated and unvaccinated patients and was not stratified by vaccination status due to the unavailability of RWE data in vaccinated patients.

Following model entry, patients were prescribed either NMV/r or usual care in the outpatient setting. To align with the dosing schedule outlined in the treatment label for NMV/r, patients were assigned one 5-day course of treatment [31]. Additionally, as all patients receive NMV/r in the outpatient setting, the model did not allow patients to receive NMV/r in the inpatient setting or receive multiple or consecutive treatment cycles of NMV/r [51]. Usual care was defined as no antiviral or monoclonal antibody therapy for the outpatient treatment of COVID-19. Although molnupiravir (Lagevrio) may be used in real-world practice to treat non-hospitalized/non-severe patients with COVID-19 at risk of progression to severe disease, it was not included in this evaluation for the following reasons. First, members of the Guideline Development Group (CDG) of the World Health Organization (WHO) made strong recommendations in favor of NMV/r and weak or conditional recommendations in favor of molnupiravir to treat this population [52]. Second, the US National Institutes of Health (NIH) listed NMV/r as the preferred therapy, followed by remdesivir; molnupiravir was only recommended when the preferred therapies were not available or clinically appropriate [53]. Lastly, NMV/r is the most widely used antiviral therapy in real-world practice in Taiwan [54,55].

Following treatment initiation, patients were subsequently grouped into one of four medical care categories based on treatment effectiveness associated with NMV/r and usual care. Based on treatment effectiveness, patients were assigned the highest level of care received in the 30-day model time horizon: (1) inpatient hospitalization, (2) emergency department (ED) visit, (3) outpatient follow-up care, or (4) no further treatment.

Patients who were hospitalized received medical care in the general ward or ICU, or received mechanical ventilation based on recent RWE analyses of HCRU for COVID-related inpatient admissions [56]. For those patients who were hospitalized, the model conservatively assumed that these patients could not be readmitted within the 30-day model time horizon due to the limited data in this geographic region.

Patients who were hospitalized may have died in the general ward or ICU (with or without mechanical ventilation) due to COVID-19 [35,48]. The primary endpoint in the EPIC-HR clinical trial was a composite of hospitalization and death; similarly, Lewnard et al. reported a composite of the number of hospitalizations and deaths associated with NMV/r, which served as the source of effectiveness data in our model [18]. However, the clinical benefit of NMV/r was assumed to directly impact reductions in hospitalizations and not death since there were no deaths reported in the NMV/r arm of the EPIC-HR trial. Instead, a separate mortality rate was applied to the hospitalized patients to demonstrate an indirect effect of NMV/r on mortality [1,18]. Patients were assumed to die at the end of inpatient hospital stay. Due to the short model time horizon of 30 days, patients were assumed to die only from COVID-19-related causes and not from natural history.

### 2.4. Model Inputs and Data Sources

All model input parameters, values, and references are provided in Table 1.

#### 2.4.1. Population Inputs

The base population of the model was representative of the Taiwanese health authority system and assumed a covered population of 23,264,640 individuals over the age of 18 in Taiwan. The most current epidemiological data, current as of this publication, were sourced where possible [1,45,46]. The 30-day average Taiwan national COVID-19 incidence (3.19%) was sourced from the Taiwan Centers for Disease Control (CDC), as of 16 November 2023 [1]. The treated population was estimated by multiplying the total number of covered lives (23,264,640) by the 30-day national incidence rate, the proportion of COVID-19 cases involving high-risk individuals obtained from the Taiwan CDC, the proportion of symptomatic cases, and the proportion of eligible patients seeking treatment (assumed as 100%) [45,46]. The proportion of symptomatic cases was sourced from a meta-analysis of the percentage of asymptomatic infections among SARS-CoV-2 Omicron variant-positive individuals [45]. This led to an eligible treatment population of 150,255.

#### 2.4.2. Clinical Inputs

Treatment effectiveness associated with NMV/r was obtained from two sources. The proportion of patients with an inpatient hospitalization was based on the primary effectiveness endpoint from Lewnard et al. (2023), as described in a prior economic model evaluating the budget impact of NMV/r from the US payer perspective [18,57]. Multiple studies evaluating the real-world effectiveness of NMV/r in the vaccinated population during the Omicron variant era of predominance exist in the peer-reviewed literature, with results consistently supporting the effectiveness of NMV/r in reducing the risk of hospitalization and mortality in patients with COVID-19 at a high risk of progressing to severe disease. We derived our effectiveness estimate from the study by Lewnard and colleagues, as it was the only study to date with detailed clinical data including time from symptom onset to COVID-19 testing [18]. The proportion of patients with an ED visit or outpatient follow-up care was based on outcomes from the EPIC-HR study [11].

The model assumed that patients who were hospitalized may have been admitted to the general ward or ICU or may have received mechanical ventilation. The proportions of patients admitted to the ICU as well as those receiving treatment via mechanical ventilation were sourced from a retrospective cohort study of hospitalized patients with COVID-19-related pneumonia from a tertiary hospital in Taiwan [49]. The proportion of patients admitted to the general ward was calculated as 100% minus the sum of proportions of ICU stay plus treatment with mechanical ventilation.

The model assumed that inpatient hospital stays did not exceed the time horizon of the model (i.e., 30 days). This assumption was based on data from a recent RWE analysis of HCRU for COVID-related inpatient admissions, which found mean (SE) inpatient length of stay of 6 (0.09) days, 21 (0.53) days, and 22 (0.51) days for the general ward, ICU, and mechanical ventilation settings, respectively [56]. These values fall within the 30-day model time horizon. The lengths of stay (LOS) for admission to the general ward and ICU and treatment with mechanical ventilation were also sourced from Yii 2024 [49].

Symptom days for hospitalized patients were assumed equal to the average LOS by highest setting of care received as reported in Yii 2024 [49]. Symptom days for outpatient management and ED visits were obtained from a US-based telephone survey among patients who tested positive for COVID-19 in the ED (but were not admitted to the inpatient setting) or in other outpatient clinics [47].

The model did not consider adverse events (AEs) because the incidence of treatment-related grade 3 or 4 AEs, or serious AEs, were similar across the NMV/r and placebo arms of the EPIC-HR study, and therefore were not expected to impact incremental results [10].

The probabilities of inpatient mortality associated with admission to the general ward and treatment with mechanical ventilation were obtained from a retrospective, observational cohort study evaluating the electronic medical records of patients with confirmed COVID-19 infection from the Hong Kong Hospital Authority [14]. The analysis was conducted among patients prescribed NMV/r during the Omicron era (January to June 2022).

### 2.5. Model Outcomes and Sensitivity Analyses

Outcomes included the total number of hospitalizations involving a general ward stay, ICU stay, and mechanical ventilation use, as well as the total number of symptom days, bed days, and ICU bed days. Additionally, mortality outcomes included the total number of inpatient hospitalization deaths, defined as the sum of the number of deaths involving the general ward, ICU, and mechanical ventilation. Outcomes in the hypothetical scenario analysis matched those in the base case analysis. Incremental results are presented, calculated as the difference between the base case analysis and the hypothetical scenario analysis.

To test the robustness of the base case scenario results against potential variation in input parameters, three separate deterministic OWSAs were conducted on total inpatient hospitalizations, total bed days, and total symptom days, respectively. In the OWSA for total inpatient hospitalizations, we varied the inpatient hospitalization rate for NMV/r, the inpatient hospitalization rate for usual care, the 30-day incidence of COVID-19 infection, and the base case NMV/r utilization rate. In the OWSA for total bed days, we varied the same parameters as the analysis on total inpatient hospitalizations, as well as general ward length of stay, ICU length of stay, the rate of ICU stays, mechanical ventilation length of stay, and the rate of mechanical ventilation use. Similarly, in the OWSA for total symptom days, we varied the same parameters as the other two OWSA analyses, as well as ED visit symptom days, the rate of ED visits for NMV/r, general ward symptom days, ICU symptom days, and mechanical ventilation symptom days. Each model parameter was varied by 20% above and below the corresponding base case value, with the exception of the 30-day hospitalization rate, which was varied according to the 95% confidence intervals for NMV/r effectiveness reported in Lewnard et al. (2023) [18]. Only the parameters affecting hospitalizations were included in the analysis. We included the base case NMV/r utilization rate in each of our OWSAs to test the robustness of model results when assuming a utilization rate that was roughly equivalent to the midpoint of the base case and the hypothetical scenario analysis.

## 3. Results

### 3.1. Base Case Analysis

**Figure 2:**
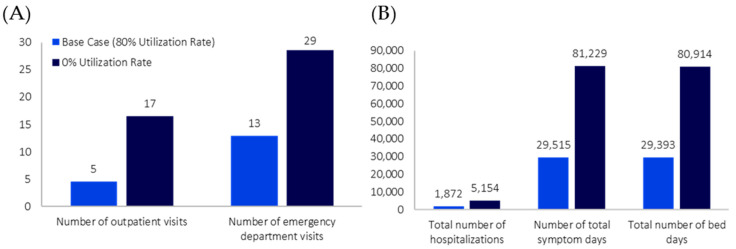
COVID-19 Model HCRU Outputs, by Varying Levels of Nirmatrelvir/Ritonavir Utilization. (**A**) Model outpatient and emergency department visit-related outputs by varying levels of nirmatrelvir/ritonavir utilization. (**B**) Model hospitalizations, total symptom dates, and bed days related outputs by varying levels of nirmatrelvir/ritonavir utilization.

**Figure 3:**
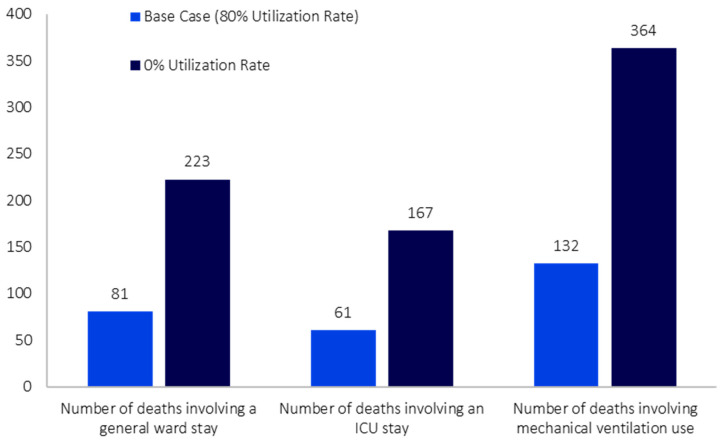
COVID-19 Model Mortality Outputs, by Varying Levels of Nirmatrelvir/Ritonavir Utilization.

After applying the 30-day incidence to the initial model population of 23,264,640 individuals, there were 742,142 individuals with COVID-19. Of those, 150,255 patients were eligible for treatment from the Taiwanese health authority perspective; the results presented in this section focus on this subgroup of the broader population with COVID-19. Assuming a treatment utilization rate of 80% for NMV/r in the base case scenario, the total number of hospitalizations (and corresponding bed days) was 1872 (29,393 bed days), including 1179 stays (21,231 bed days) in the general ward, 356 stays (4624 bed days) in the ICU, and 337 hospitalizations (3538 bed days) involving mechanical ventilation use (Figure 2). The numbers of outpatient visits and ED visits were 5 and 13, respectively (Figure 2). The total number of symptom days was 29,515 (Figure 2). The total number of inpatient deaths was 274, including 81 deaths involving stays in the general ward, 61 deaths involving stays in the ICU, and 132 deaths involving mechanical ventilation use (Figure 3).

### 3.2. Hypothetical Scenario Analysis

In the hypothetical scenario (i.e., absence of NMV/r), the total number of hospitalizations increased by 175% compared to the base case analysis (difference: 3282 hospitalizations). This pattern of increased HCRU in the hypothetical scenario extended to the number of hospitalizations involving stays in the general ward and ICU (differences: 2067 and 623 hospitalizations, respectively), the number of hospitalizations involving mechanical ventilation use (difference: 591 hospitalizations), and the total number of bed days (difference: 51,521 bed days), each increasing by 175% when a treatment utilization rate of 0% was used for NMV/r (Figure 2). Similarly, the number of outpatient and ED visits increased by 267% and 121%, respectively (differences: 12 and 16 visits) (Figure 2). The total number of symptom days also increased by 175% (difference: 51,714 symptom days) (Figure 2). In the hypothetical scenario, the total number of deaths increased by 175% (difference: 480 deaths); the number of deaths by highest setting of care received followed a similar trend (Figure 3).

### 3.3. Sensitivity Analysis

**Figure 4:**
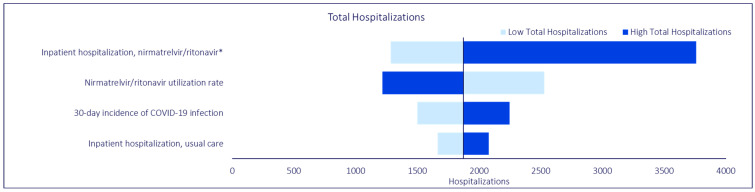
COVID-19 Model One-Way Sensitivity Analysis: Total Hospitalizations. * The low and high estimates were based on reported variance from Lewnard et al. [18]. All other parameters are varied +/−20% from base case.

**Figure 5:**
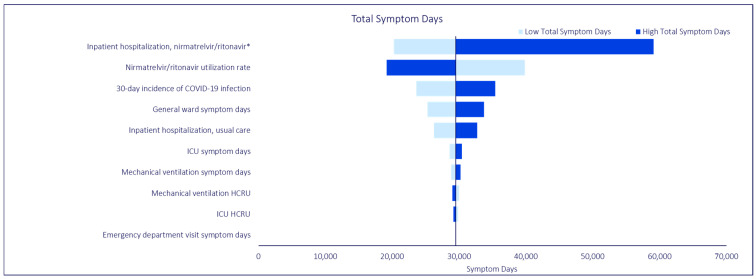
COVID-19 Model One-Way Sensitivity Analysis: Total Symptom Days. * The low and high estimates were based on reported variance from Lewnard et al. [18]. All other parameters are varied +/−20% from base case.

**Figure 6:**
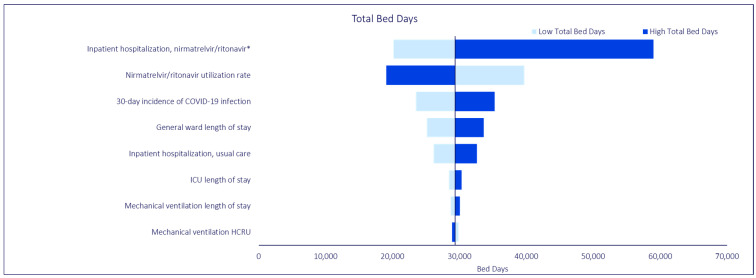
COVID-19 Model One-Way Sensitivity Analysis: Total Bed Days. * The low and high estimates were based on reported variance from Lewnard et al. [18]. All other parameters are varied +/−20% from base case.

OWSAs were conducted to evaluate the sensitivity of the base case analysis to variations in model input values. All OWSAs generated model findings that continued to demonstrate the beneficial impact of NMV/r, despite variations in model input values, which were consistent with findings from the base case analysis. Regardless of key model outcome (i.e., total inpatient hospitalizations, total bed days, total symptom days), the model was most sensitive to variations in the inpatient hospitalization rate for NMV/r, base case NMV/r utilization rate, and 30-day incidence of COVID-19 infection. In the OWSA for total bed days, general ward length of stay, the inpatient hospitalization rate for usual care, and ICU length of stay were also influential parameters. Similarly, in the OWSA for total symptom days, general ward symptom days, the inpatient hospitalization rate for usual care, and ICU symptom days were influential parameters. The findings are summarized in Figure 4, Figure 5 and Figure 6.

## 4. Discussion

### 4.1. Overview

In this analysis from the Taiwanese health authority perspective, a treatment utilization model was implemented to estimate the clinical benefits of sustained use of NMV/r associated with mild-to-moderate COVID-19 among vaccinated and unvaccinated individuals at high risk of progression to severe disease and with access to treatment. Based on real-world clinical effectiveness data associated with NMV/r, our findings indicate that sustaining the current utilization of NMV/r will avoid a potential 175% increase in both hospitalizations and deaths if NMV/r is no longer made publicly available. This translates to a significant impact on the health system, by potentially freeing up 51,521 bed days.

### 4.2. Comparison to the Literature

To our knowledge, there are no studies assessing the healthcare resource use and mortality implications of NMV/r utilization from the Taiwanese health authority perspective. Multiple models have been developed from the US payer perspective that are largely consistent with our model structure [57,58] (i.e., healthcare resource use by highest setting of care received) and employ similar primary effectiveness inputs. However, there are key differences between these published models and our model, including time horizon and geographical location.

Health system-specific data, specifically on length of stay related to inpatient hospitalizations, are a key driver in differences between our model and those previously developed. Specifically, while the average length of stay for mechanical ventilation in Taiwan is similar to that in the US (23.5 days in Taiwan (estimated as the sum of time spent in the ICU and on mechanical ventilation) vs. 22 days in the US), time spent in the general ward and ICU differs (general ward = 18 days in Taiwan vs. 6 days in the US; ICU = 13 days in Taiwan vs. 21 days in the US), highlighting the difference in standard of care practices across the two regions. Other differences include the inclusion of other antivirals in addition to NMV/r. For example, two recent analyses by Sandin and colleagues included combinations of molnupiravir, sotrovimab, and remdesivir [57,58]. Similarly, a model conducted by the Institute for Clinical and Economic Review (ICER) included molnupiravir and fluvoxamine [31]. For the reasons stated above in the Section 2 (i.e., WHO recommendations, US NIH preferential considerations, Taiwanese treatment practices), our analysis focused on an evaluation of NMV/r.

### 4.3. Limitations

The clinical benefits of NMV/r have been maintained regardless of the setting (clinical trial; real world) or predominant variant (Delta; Omicron). Despite this, it is possible that the effectiveness of NMV/r may change over time. The effectiveness of NMV/r used in this model was based on real-world data evaluated among patients treated during the Omicron era. New subvariants of Omicron are circulating (e.g., EG.5, FL.1.5.1, XBB.1.16.6, XBB.1.16.11) [59], and the effectiveness of NMV/r has been assumed to be consistent across these subvariants. To address this assumption, the inpatient hospitalization rate for NMV/r was varied by ±20% in OWSAs.

Our model used data from the EPIC-HR clinical trial to inform the proportion of patients with an ED visit or outpatient follow-up care. While this integrates clinical trial data with those from real-world studies (i.e., the Lewnard analysis for inpatient hospitalizations), real-world studies assessing the impact of NMV/r on ED or outpatient resource use are limited [18]. As noted above, real-world analyses of the effectiveness of NMV/r on inpatient hospitalizations have largely confirmed the findings from the EPIC-HR trial; therefore, we would anticipate the same for ED or outpatient resource use if available, minimizing implications for model findings.

Within the EPIC-HR clinical trial and the Lewnard analysis, mortality was assessed as part of a composite endpoint (i.e., reduction in 30-day hospitalizations or mortality). Since the trial results did not disaggregate 30-day hospitalizations from mortality, our model applied the same probability of death, by inpatient setting, to the NMV/r and usual care cohorts. The difference in mortality outcomes in the model, therefore, was driven by the number of patients with an inpatient hospitalization, which was an indirect artifact of the clinical benefit of NMV/r.

Furthermore, patient mortality was assumed to occur only from COVID-19-related causes, and not from all-cause reasons, given the short duration of the model time horizon (i.e., 30 days). Excluding all-cause mortality from our model is unlikely to significantly impact outcomes, as all-cause mortality would have been applied to both treatment cohorts at the same rate. As such, any difference in mortality was truly driven by the difference in COVID-19-related inpatient hospitalizations between the NMV/r and usual care cohorts. Additionally, patients who died were assumed to do so at the end of the inpatient hospital stay. It is common in health economic modeling for events and costs to be applied at either the start or end of a model cycle. In this case, we applied death at the end of the inpatient hospitalization in order to ensure that all potential healthcare resource use associated with the inpatient hospitalization was appropriately accounted for within the model. We consider this to be a conservative approach given it likely overestimates the burden associated with mortality; for example, by attributing the full hospitalization healthcare resource use to a patient who dies, the model assumes that all symptom days related to the hospitalization are incurred, even if the patient may not have utilized all resources had they survived.

Our model did not stratify treatment cohorts (i.e., NMV/r, usual care) by vaccination status. While the Lewnard analysis included both patients who had and had not received COVID-19 vaccinations, outcomes in the Lewnard analysis were not stratified based on vaccination status. For this reason, we assumed a homogenous population including a mix of vaccinated and unvaccinated patients. The data used in our analysis are aligned with this approach and reflect the current population mix in Taiwan. Furthermore, model results are reported as homogenous outcomes. When considering future research, it would be beneficial to understand the implications of vaccination status for healthcare resource use and costs for patients with COVID-19, especially those at high risk of progressing to severe disease.

Our model took a conservative approach by focusing on the acute phase of disease (i.e., 30 days) and did not project to a one-year time horizon (or beyond). The current evidence indicates that long-term effects of COVID-19 may affect the heart, lungs, kidney, and brain, thereby increasing the risk of developing secondary complications and diseases, which occur more often in people who had severe COVID-19 [60,61]. Moreover, potential long-term impacts of COVID-19, such as Long COVID and post-ICU discharge effects, may result in substantial long-standing clinical and economic outcomes. Focusing on a short-term time horizon precluded us from extrapolating short-term clinical benefits of NMV/r to a long-term time horizon, thereby substantiating the conservative modeling approach.

Finally, our model did not investigate the potential spillover effect of treatment with NMV/r. This conservative approach ignores any population-level indirect effects (i.e., reductions in transmission of COVID-19), as mentioned in the analysis by Bai and colleagues, as well as the reduction in risk of other morbidities, such as cardiovascular disease [27,61,62].

### 4.4. Implications

The detrimental health and economic effects of the COVID-19 pandemic have been well documented in various publications and throughout the press. The acute infection period can be especially burdensome, characterized by the potential for progression to severe disease, expensive hospitalizations, and receipt of mechanical ventilation to stabilize respiratory failure. For those patients with mild-to-moderate COVID-19 and high risk for progression to severe disease, effective treatment options exist to prevent disease worsening and thus avoid a high clinical burden for patients, caregivers, the health system, and society as a whole.

Our analysis demonstrates that a sustained level of NMV/r use will likely avoid increases in HCRU, including a substantial impact on available hospital beds and mortality, irrespective of vaccination status, from the Taiwanese health authority perspective. Reducing the use of healthcare resources due to COVID-19 will contribute to cost containment by placing less financial strain on the Taiwanese health system as well as on patients in the form of out-of-pocket healthcare costs. Moreover, healthcare resources that would have been used to manage hospitalized COVID-19 cases can be redirected for use by patients with other conditions and diseases.

Given the real-world clinical effectiveness associated with NMV/r, continuously stockpiling the current supply of NMV/r will likely maintain a considerable benefit in Taiwan. These findings are grounded in the clinical effectiveness of NMV/r and are corroborated by other economic modeling analyses of NMV/r [56,57,58] conducted in various geographic regions. However, the magnitude of clinical and economic benefits of NMV/r will depend on specific healthcare management strategies, resource availability and utilization, and per unit direct medical costs (in addition to local epidemiological estimates) in different countries. Thus, caution should be exercised when generalizing these findings to healthcare settings with differing COVID-19 treatment protocols and resource utilization.

## 5. Conclusions

Based on the findings from this analysis, sustained utilization of NMV/r reduces the clinical burden of COVID-19 through the reduced incidence of COVID-19-related hospitalizations, outpatient care, and deaths from the Taiwanese health authority perspective. Maintaining, or increasing, the widespread availability of outpatient therapy with NMV/r for mild-to-moderate COVID-19 during the acute infection period, therefore, may avoid adverse outcomes.

## Figures and Tables

**Table 1:** Model Input Parameters and Values.

Model Input Parameter	Base Case Value (OWSA Low Value; OWSA High Value)		Reference
*Population Inputs*			
Population size, *n*	23,264,640		[44]
30-day incidence of COVID-19 infection, %	3.19 (2.55; 3.83)		[1]
Proportion of COVID-19 cases involving high-risk individuals, %	31.60		[46]
Proportion of cases that are symptomatic, %	64.07		[45]
Proportion of eligible patients seeking treatment, %	100.00		Assumption
*Clinical Inputs*	*Nirmatrelvir/Ritonavir*	*Usual Care*	
Proportion with highest setting of care, %			
Outpatient management	0.001 (0.0008; 0.0012)	0.011 (0.009; 0.013)	[11]
Emergency department	0.006 (0.005; 0.007)	0.019 (0.015; 0.023)	[11]
Inpatient hospitalization	0.70 (0.21; 2.27)	3.43 (2.74; 4.12)	[18]
Proportion of inpatient resource utilization by setting, %			
General ward	63.0		Calculation
ICU	19.0 (15.2; 22.8)		[49]
Mechanical ventilation	18.0 (14.4; 21.6)		[49]
Proportion of inpatient hospitalizations resulting in death, %			
General ward	6.9		[48]
ICU	17.1		[35]
Mechanical ventilation	39.2		[48]
Length of stay, days			
General ward	18.0		[49]
ICU	13.0		[49]
Mechanical ventilation	10.5		[49]
Symptom days, by highest setting of care and respiratory support received			
Outpatient management	7.0		[47]
ED visit	7.0		[47]
Inpatient hospitalization/level of respiratory support received			
General ward	18.0		[49]
ICU	13.0		[49]
Mechanical ventilation	10.5		[49]

ED: emergency department; ICU: intensive care unit; OWSA: one-way sensitivity analysis.

## Data Availability

The data presented in this study are readily available in the published literature; full citations for each data input are presented in the reference section of this manuscript.
